# Editorial: Organic amendments: microbial communities and their role in plant fitness and disease suppression

**DOI:** 10.3389/fpls.2023.1213092

**Published:** 2023-07-06

**Authors:** Iakovos S. Pantelides, Ioannis A. Stringlis, Omri M. Finkel, Jesús Mercado-Blanco

**Affiliations:** ^1^ Department of Agricultural Sciences, Biotechnology and Food Science, Cyprus University of Technology, Limassol, Cyprus; ^2^ Laboratory of Plant Pathology, Agricultural University of Athens, Athens, Greece; ^3^ Department of Plant and Environmental Sciences, Institute of Life Sciences, The Hebrew University of Jerusalem, Jerusalem, Israel; ^4^ Department of Soil and Plant Microbiology, Estación Experimental del Zaidín, Consejo Superior de Investigaciones Científicas (CSIC), Granada, Spain

**Keywords:** organic amendments, microbial communities, plant growth promotion (PGP), disease suppression, nutrient cycling

Organic amendments have long been used to improve soil fertility, structure, and water-holding capacity, thereby promoting plant growth and productivity (Bailey and Lazarovits, 2003; Larney and Angers, 2012; Meghvansi and Varma, 2015). In addition to their physical and chemical benefits, organic amendments also play a critical role in shaping the soil microbial communities, which are key drivers of plant fitness and health (Pascale et al., 2020). Recent advances in high-throughput sequencing and omics technologies have shed new light on the complex interactions between organic amendments, microbial communities, and plants. Understanding the mechanisms by which organic amendments impact soil microbial communities and plant health is critical for developing sustainable agricultural practices.

In this Research Topic, we gathered the latest research on the effects of organic amendments on soil microbial communities, nutrient cycling, plant growth promotion, and disease suppression. The seven research articles in this Research Topic cover a wide range of topics, including the impact of various organic amendments on soil microbes and their functions and the mechanisms underlying these interactions.

One of the main themes of this Research Topic is the use of organic amendments to enhance soil microbial diversity and activity, which in turn can improve plant growth and health. Zhang M. et al. investigated the effect of biochar on soil nutrient content, enzymatic activity, and fungal community structure in yellow soil typical of karst areas. Three biochar levels (0%, 1.0%, and 4.0%) showed that biochar increased pH, organic matter, total nitrogen, available phosphorus, and potassium, but decreased microbial biomass. High-application-rate biochar increased the abundance of certain fungal genera while impeding harmful pathogen growth and increasing the abundance of beneficial fungi. Biochar positively impacted land productivity and the micro-ecological environment, with available potassium being a crucial factor.

Another study by De Tender et al. investigated how biochar affects the resistance of strawberry fruits and leaves to the pathogenic fungus *Botrytis cinerea*. The study found that biochar treatment increased the resistance of strawberry fruits, but not leaves, which was linked to changes in the rhizosphere microbiome characterized by the rise of beneficial bacteria. These changes in the rhizosphere microbiome might have contributed to the enhanced fruit resistance. Overall, the study highlights the potential of biochar to improve plant resistance to fungal pathogens through its effects on the rhizosphere microbiome.

While organic amendments have been shown to positively impact soil health and plant fitness, the mechanisms underlying these effects are still poorly understood. Wang et al. investigated the impact of *Bacillus subtilis* bioorganic fertilizer on soil microbial communities and plant growth in *Brassica chinensis* L. The authors found that bioorganic fertilizer application increased plant height and biomass, as well as soil available potassium and pH. Using high-throughput sequencing, the authors examined the bacterial and fungal communities in the soil and found that their diversity was reduced in the bioorganic fertilizer treatment compared to the control group. Additionally, the authors found that some beneficial bacteria and fungi were enriched in the bioorganic fertilizer treatment, and that the application of bioorganic fertilizer enhanced the function of mineral element metabolism and absorption. These findings suggest that *Bacillus subtilis* bioorganic fertilizer may be a promising organic amendment for improving soil health and plant productivity.

Two other studies in this Research Topic explored the effects of replacing chemical fertilizers with organic alternatives or their mixture on soil health, crop productivity, and bacterial community diversity. In the first, Zhang H. et al. found that substituting a chemical fertilizer with biogas slurry in an apple orchard produced an increase in bacterial community diversity and abundance, with a higher proportion of beneficial bacteria and a lower abundance of harmful bacteria. This substitution also increased soil enzyme activities, including those involved in nitrogen, potassium, and carbon cycling. The results suggest that biogas slurry can positively affect the soil bacterial community, leading to improved soil health and plant growth in apple orchards. In the second study, Iqbal et al. investigated the effects of partially substituting organic fertilizer with chemical fertilizer in a paddy field and found that it improved soil biochemical attributes and resulted in higher rice yields compared to using organic fertilizer alone. The bacterial community diversity was also restored by the partial substitution, indicating a potential positive impact on soil health. This suggests that partial substitution of organic fertilizer with chemical fertilizer may be a feasible option to improve soil quality and crop productivity while maintaining bacterial community diversity in paddy fields.

Another theme of this Research Topic is to understand the intricate interactions among plants, soil, and microbial communities that enhance crop quality and yield. Jiang et al. investigated the impact of long-term organic fertilizer addition on soil extracellular enzyme activities and tobacco quality in a tobacco-maize rotation system. Organic fertilizer additions improved tobacco yield and quality as well as soil extracellular enzyme activities. Pig dung was found to be the most effective organic fertilizer among those tested, leading to higher levels of soil organic matter, total organic carbon, total phosphorus, and available phosphorus compared to oil cake and straw. Soil extracellular enzyme activities were positively related to tobacco yield and quality. The study recommends combining pig dung with chemical fertilizers to enhance soil nutrients and tobacco quality.

The study by Gaete et al. investigated the effect of water deficit-tolerant and susceptible *Solanum lycopersicum* cultivars on their rhizosphere microbiome, and compared it with plant-free soil microbial communities. The researchers found that the tomato cultivars had a significant effect on the alpha and beta diversity of their bacterial rhizosphere communities, but not on their fungal communities. Water deficit increased alpha diversity in both bacteria and fungi in the susceptible rhizosphere but not in the tolerant rhizosphere cultivar, indicating a buffering effect of the tolerant cultivar on its rhizosphere microbial communities. Furthermore, the tolerant cultivar had a more efficient microbial community in response to water deficit, with increased cooperation within kingdoms. Specific bacteria and fungi in the tolerant cultivar may contribute to its water deficit tolerance.

Overall, the articles in this Research Topic underscore the significance of organic amendments, organic and bioorganic fertilizers, as well as microbial communities in promoting plant fitness and disease suppression. These findings have important implications for how we develop and apply sustainable strategies in agriculture, as they provide new insights into the use of organic amendments and microbial inoculants to enhance soil health and productivity, while reducing reliance on synthetic fertilizers and pesticides. Still, more research is needed to understand the mechanisms by which organic amendments impact soil microbial communities and plant health. We hope that this Research Topic will inspire future research in this exciting and rapidly evolving field, and stimulate new ideas and collaborations among researchers, farmers, and other stakeholders in the agricultural community.

## Author contributions

The authors defined together the content of this Research Topic and all participated in the editing process. All authors made critical contribution to the writing of this editorial and approved it for publication.
